# The different effects of a sensorimotor grounding on AoA between bilingual concepts

**DOI:** 10.3389/fnhum.2024.1387674

**Published:** 2024-05-10

**Authors:** Jin Xue, Dongcheng Xie, Xinyi Lu, Zihan Niu, Fernando Marmolejo-Ramos

**Affiliations:** ^1^School of Foreign Studies, University of Science and Technology Beijing, Beijing, China; ^2^University of South Australia Online, Adelaide, SA, Australia

**Keywords:** AoA, sensorimotor, grounding, L1 Chinese, L2 English

## Abstract

**Introduction:**

Psycholinguistic studies have argued for the age of acquisition (AoA) of words as a marker of concept learning, showing that the semantic features of concepts themselves influence the age at which their labels are learned. However, empirical evidence suggests that semantic features such as imageability and linguistic phenomena such as frequency do not adequately predict AoA. The present study takes the developmental approach of embodied cognition and investigates the effects of sensorimotor experiences on the ease of acquisition of the concept acquired in bilinguals. Specifically, we investigated (1) whether the sensorimotor experience can explain AoA beyond frequency; (2) and whether these patterns are consistent across L1 Chinese and L2 English.

**Methods:**

We conducted sensorimotor rating measures in both Chinese and English on 207 items in which Chinese-English bilingual adults were requested to evaluate the extent to which they experienced concepts by employing six perceptual senses and five effectors for actions located in various regions of the body. Meanwhile, data on AoA and frequency were collected.

**Results:**

The present study showed the sensorimotor experience was closely linked with AoAs in both languages. However, the correlation analysis revealed a trend of higher correlations between AoAs for the same concepts and L1 Chinese, relative to L2 English for the present Chinese-English bilinguals. Importantly, the hierarchical regression analysis demonstrated that after controlling for frequency, sensorimotor experience explained additional variance in L1 AoA. However, L2 sensorimotor experience did not explain the variance in L2 AoA. Sensorimotor experience explained more share of variance in L1 AoA but frequency accounted for more variance in L2 AoA.

**Discussion:**

The findings suggest that concept acquisition should consider the grounding in appropriate sensorimotor experience beyond linguistic phenomena like frequency.

## 1 Introduction

Psycholinguistic research has investigated the age of acquisition (AoA) of words as an indicator of concept learning. One traditional approach to studies on AoA is to investigate the impact of linguistic factors, such as input frequency (Ambridge et al., 2015; Roy et al., 2015), or semantics features like concreteness or imageability (Bird et al., 2001). Contrastively, embodied theories of cognition propose that concepts can be grounded in the embodied experience. For instance, the measure of body-object interaction (BOI) can be added to the traditional measures like frequency, imageability, and valence in explaining AoA (Thill and Twomey, 2016). BOI refers to the perceived ease with which the human body can physically interact with the object referred to by a word. Hence, this line of research highlights the importance of embodied information in understanding the effect of AoA on concept acquisition. The present study aims to investigate how sensorimotor strength influences the ease of concept acquisition among bilingual individuals.

It is well-established that input frequency has a significant impact on the AoA of words (Blackwell, 2005; Goodman et al., 2008; Ambridge et al., 2015). The independent impact of input frequency on the age at which words are acquired has been empirically demonstrated in different word categories like nouns and function words (Goodman et al., 2008), verbs (Theakston et al., 2004), and adjectives (Blackwell, 2005). According to the review article (Ambridge et al., 2015), language acquisition is influenced by frequency-sensitive learning mechanisms, regardless of other theoretical assumptions. The article suggests that high-frequency forms are acquired early and that the frequency of input significantly contributes to the variance of AoA, even when considering other factors in the regression model.

Evidence further suggests that the frequency effect on AoA is modulated by semantic status. This is supported by the observation that words used in particular spatial, temporal, and linguistic contexts are produced at an earlier stage (Roy et al., 2015). The mental image evoked by a word (Ma et al., 2009) and the perceptibility of a concept (Bird et al., 2001; Brysbaert et al., 2013) are semantic features that affect the learning of corresponding labels. It is argued that frequency and imageability are the most significant predictors of rated age of acquisition, despite the contribution of word length, familiarity, and concreteness to the measure (Bird et al., 2001). The involvement of the senses in understanding concepts is often considered evidence for a grounded or embodied view.

Under the grounded cognition framework, sensorimotor knowledge plays a crucial role in lexical processing (Barsalou, 2008). A growing body of evidence supports the idea that concepts are grounded in sensorimotor experience (Siakaluk et al., 2008; Klepp et al., 2019). For example, in studies such as semantic categorization and semantic decision tasks (Siakaluk et al., 2008), responses were found to be faster and more accurate for words with high BOI ratings. These findings suggest that BOI facilitates both semantic feedback and processing. They are consistent with the grounded cognition framework, which emphasizes the incorporation of sensorimotor interactions with the environment into semantic knowledge. Furthermore, neuroimaging evidence discovered a verb-motor priming effect in both behavioral and neurophysiological measures (Klepp et al., 2019), which provides insight into the involvement of sensorimotor brain regions in language processing and demonstrates their flexibility. This finding supports the concept of embodied and grounded cognition, suggesting that cognitive systems are not merely superficially embodied through sensorimotor interactions with the environment. Instead, cognitive systems are truly embodied, reflecting a deep integration of mind and body (Stapleton, 2013). Supported by convergent clinical and cognitive neuroscience data, a more recent framework called controlled semantic cognition suggests that semantic cognition depends on two main neural systems: representation and control (Ralph et al., 2017). In the representation system, concepts are formed through the hub-and-spoke method. The hub is localized in the bilateral anterior temporal region, while the spoke is localized in modality-specific association cortices spread throughout the cortex. The control system, on the other hand, is implemented in a distributed neural network that includes frontal and temporoparietal regions. This system supports executive mechanisms that regulate how activation spreads through the network in semantic representation.

Research on the grounding of concepts was not typically restricted to the first language (L1). There are compelling reasons to consider the issue from a bilingual perspective. First and foremost, any mechanistic account of concept grounding makes the direct prediction that whatever mechanism is proposed in the L1 can be subserved when bilinguals use the concepts in the second language (L2). For instance, a recent study explored the impact of embodied morphological instruction on Chinese-English bilinguals (Guan and Meng, 2022). It compared three learning methods involving physical interaction with word roots (handwriting, dragging, gesturing) with a control condition. The results showed that embodied information facilitated concept learning in their L2 English. Secondarily, physical and cognitive development may be a crucial component of explanatory accounts of cognitive mechanisms in bilinguals. Although the influence of sensorimotor experience on learning persists across the lifespan (Kontra et al., 2012), there should be dramatic differences when learners acquire concepts in different language contexts at different stages of the lifespan. Research on the acquisition of human concepts and words supports that the effects of frequency vary as development progresses (Goodman et al., 2008). Therefore, the influence of frequency on the acquisition of vocabulary is shaped by an intricate interplay between category, modality, and developmental stage. Thus, it makes academic sense to ask whether the contribution of sensorimotor experience to AoA of bilingual concepts is consistent across L1 and L2 when bilinguals acquire the two languages consecutively.

The literature on bilingualism encompasses a substantial body of research focused on the grounding of bilingual concepts (Bird et al., 2001; Goodman et al., 2008; Ma et al., 2009; Kontra et al., 2012; Guan and Meng, 2022). Several studies have found similar effects of groundability in L1 (Ma et al., 2009; Koster et al., 2018). In particular, Koster et al.’s (2018) study effectively replicated prior simulation studies analyzing the impact of object size and orientation on bilingual individuals. The findings further validate the concept that the intrinsic characteristics of objects are simulated in the second language (L2). In the study by Ma et al. (2009), Chinese adults were asked to rate the imageability of Chinese words. The results showed that imageability ratings accurately predicted AoA for both nouns and verbs in Chinese. Additionally, imageability appeared to have a unique contribution to the variance in AoA of verb learning, independent from input frequency, which aligns with previous research conducted in English. It is also important to highlight that this study revealed verbs obtained higher imageability ratings in L1 compared to L2. These results suggest the varying impact of sensorimotor strength in the acquisition of bilingual concepts.

Some other studies have observed a similar reduction or absence of grounding effects in L2 (Sheikh and Titone, 2015; Chen et al., 2020; Norman and Peleg, 2021). For instance, bilingual participants completed a task in both their L1 and L2, where they read sentences and immediately decided whether a pictured object had been mentioned in the preceding sentence (Norman and Peleg, 2021). Results showed that responses were significantly faster when the shape of the object in the picture matched the sentence-implied shape, but only in the L1, and only when the L1 block was performed before the L2 block. The findings indicate that there is a variation in embodied information between L1 and L2. In an eye-tracking study (Sheikh and Titone, 2015), it was observed that positive words were read more rapidly than neutral words in the L2 across first-pass reading time measures. However, this emotional advantage was not seen for negative words in the earliest measures. Additionally, negative words were influenced by concreteness, frequency, and L2 proficiency in a manner similar to neutral words. This phenomenon is attributed to the selective use of experiential sources to establish a foundation for L2 semantics. Similarly, a study on trilingual adults proficient in Cantonese, Mandarin, and English used delayed sentence-picture verification tasks to investigate multilingual perceptual representations (Chen et al., 2020). Results showed a significant match effect in L1 Cantonese, but not in highly proficient L2 Mandarin or low proficient L3 English. The authors argued in favor of the distributed conception view, suggesting that L2 and L3 are associated with fewer perceptual symbols than L1. However, the researchers ruled out the possibility that language proficiency levels have an impact on the aforementioned perceptual representations in delayed tasks, based on the similar pattern observed in highly proficient L2 and low proficient L3. The results indicate that concepts are less embodied in L2 than in L1 for bilingual individuals.

Given the divergent findings mentioned above, further research is needed to determine how sensorimotor experience contributes to the AoA of concept acquisition in bilingual contexts. This study aimed to combine the frequency and sensorimotor variables, which have been shown individually to influence AoA, in a single analysis. The primary aim was to test whether sensorimotor variables can provide additional insight beyond the frequency factor, thus highlighting the importance of identifying and testing sensorimotor grounding factors in bilingual learning. In addition, this study sought to investigate whether the effect of sensorimotor grounding on AoA was consistent across L1 and L2. Thus, two research questions were addressed in this study:

1. To what extent can the grounding of concepts in sensorimotor experience explain AoA of word concepts, beyond the influence of frequency?
2. Did the sensorimotor strengths for concept learning remain consistent across both L1 Chinese and L2 English?

It was predicted that the degree to which concepts are rooted in sensorimotor experience would influence the AoA of word concepts. It was also predicted that the influence of sensorimotor experience on L2 English acquisition would be limited compared to L1 Chinese. Additionally, it was predicted there was an interaction between L1 and L2 sensorimotor experience, which was expected to contribute to bilingual concept learning. It was not our intention to provide a comprehensive analysis of the influence of sensorimotor and linguistic factors on AoA. However, to the best of our knowledge, the present study was the first to combine frequency and sensorimotor variables to elucidate the concept of AoA in a bilingual context.

## 2 Materials and methods

### 2.1 Materials

The stimuli consisted of two separate lists of 207 words of the same concepts, one in L1 Chinese and the other in L2 English. The English word list was carefully chosen from the Lancaster Sensorimotor norms (Lynott et al., 2020), which encompass measurements of sensorimotor strength for a vast range of concepts – a total of 39,707 – spanning six different ways of perceiving things: touch, hearing, smell, taste, vision, and interoception. Moreover, these norms also cover five distinct aspects in which actions are performed: mouth/throat movements, hand/arm movements, foot/leg movements, head movements (excluding mouth/throat movements), and torso movements. Consequently, these norms are highly advantageous for researchers working in a variety of fields, such as psycholinguistics, grounded cognition, cognitive semantics, knowledge representation, machine learning, and big data analyses of linguistic and conceptual representations.

Since the focus of this study was on Chinese native learners of English, English words selection had to meet certain criteria: they had to be easily recognizable by college-level L2 English learners, so these words had to have 100% recognition rate in the variables “Percent_known.action” and “Percent_known.perfect” in the Lancaster Sensorimotor Norms (Lynott et al., 2020). As sensorimotor was the key research interest of the present study, we categorized these words into five groups (≤3, <4, <5, <6, ≥6) according to the rating of Minkowski 3 strength in the Norms. According to the summary descriptives of the dataset, Minkowski 3 strength represents an optimal single-variable composite of the 11-dimension sensorimotor profile, thus acting as a powerful semantic facilitator in lexical decision performance (Lynott et al., 2020). Subsequently, 80 words were randomly selected from each category, forming an initial set of 400 English words. These words were then translated from English to Chinese by the second author by referencing the Collins dictionary. The next step involved obtaining ratings from 12 undergraduate students at a university in Beijing. These students were asked to indicate their familiarity with each English word (1 for knowing, 0 for not knowing) and to assess the accuracy of the provided Chinese translations. If they disagreed with a translation, they were requested to suggest a more appropriate Chinese translation.

Finally, a total of 207 English words, with a recognition accuracy of a minimum of 85% of Chinese native speakers of English, along with their corresponding Chinese equivalents, were chosen as the final experimental stimuli (see https://osf.io/kpqjv/). The word lists exhibited similarities with regard to the Lancaster Sensorimotor Norms. These words encompassed various significant syntactic categories such as nouns (*N* = 121), verbs (*N* = 42), adjectives (*N* = 23), and adverbs (*N* = 21), covering a broad range of concepts including but not limited to foods, animals, emotions, sports, taboo words, professions, and colors. Subsequently, we divided the complete item set into five word lists, each containing 39 to 42 test items, for both English and Chinese languages, respectively. Following established research practices (Lynott et al., 2020), a set of five calibrator words were consistently presented at the beginning of each item list. This was done to acquaint participants with clear and definitive examples of items encompassing different sensorimotor strength across different dimensions. These criteria included low sensorimotor strength across all modalities (illustrated by the word “dream”), and high strength across multiple modalities (conveyed by “chair”).

### 2.2 Participants

A total of 137 participants [*M*_age_ = 24.29; *SD* = 3.64; range = (16, 35); *N*_female_ = 97; *N*_male_ = 40] completed 168 surveys on Chinese words using the professional platform called ‘Wenjuanxing’ for data collection questionnaire (https://www.wjx.cn/). Among them, 6 (4.4%) were high school students, 89 (65%) were college students, and 42 (30.6%) were postgraduates. Similarly, a total of 118 participants [*M*_age_ = 23.85; *SD* = 3.38; range = (19, 34); *N*_female_ = 88; *N*_male_ = 30] completed 164 surveys on English words. Of these, 67 (56.8%) were college students and 51 (43.2%) were postgraduates. Both groups had Chinese Mandarin as their L1 and English as their L2, and were matched in age, *F* (1, 353) = 1.01, *p* = 0.32. The two groups were also matched on the onset age for receiving formal education in L1 Chinese [for Chinese wordlists participants, *M* = 4.30, *SD* = 2.21; for English wordlists participants, *M* = 4.26, *SD* = 2.12; *F* (1,253) = 0.02, *p* = 0.89], and on the onset age for receiving formal education in L2 English [for Chinese wordlists participants, *M* = 8.55, *SD* = 2.46; for English wordlists participants, *M* = 8.48, *SD* = 2.51; *F* (1,253) = 0.04, *p* = 0.84]. 57 participants rating the Chinese wordlists (41.6%) and 42 participants rating the English wordlists (35.6%) reported that besides Mandarin they spoke other languages or dialects like Cantonese, Min, Hakka, and Gansu dialects. Forty-six participants rated both Chinese and English word lists. On average, each participant completed an average of 1.23 Chinese word lists and 1.39 English word lists. Participants were asked to rate all modalities of perceptual strength and effectors of action strength. They could complete multiple wordlists but were not allowed to repeat any list in Chinese or English. Specifically, for a given list of words, a participant rated either the Chinese or English list but not both simultaneously. The participants were self-selected and only experienced users of online questionnaire platforms were recruited. Participants were also asked to report AoA for the specific words they rated. Demographic information of the participants was also collected during the survey.

### 2.3 Data collection procedure

We utilized Wenjuanxing, a professional online questionnaire platform in China, to create a standardized survey based on the procedures outlined in previous research (Lynott et al., 2020). Prior to the survey, participants were provided with an information sheet and required to give their informed consent to proceed with the study. Following this, participants were given detailed instructions, explaining that they would need to rate their experience of everyday concepts using six perceptual senses and five action effectors from different parts of the body. The instructions emphasized that there were no right or wrong answers, and participants were encouraged to rely on their judgment. The Likert rating scales ranged from 0 (indicating no experience with the specific sense or action) to 5 (indicating a substantial level of experience). To help distinguish between the five distinct effectors (Foot/leg; Torso; Hand/arm; Head; Mouth/throat) when rating the action strength, images of a human virtual entity were presented, highlighting the specific body part associated with each effector. Participants were also instructed to select the “Do not know it” option if they encountered unfamiliar words and proceed to the next item.

To assess perceptual strength, participants were asked to rate the extent to which they experienced “WORD” (e.g., “seller”). Each item was presented individually on a separate rating screen, along with a brief introduction. The rating scales are displayed below, corresponding to the perceptual modalities being investigated (Visual, Haptic, Auditory, Gustatory, Olfactory, and Interoceptive). Each scale was specifically labeled as “By feeling through touch,” “By hearing,” “By sensations inside your body,” “By smelling,” “By seeing,” and “By tasting.” For action strength, participants were asked to rate the extent to which they experienced the “WORD” by performing an action with a specific body part. Five rating scales were provided, each corresponding to an action effector: “Foot/leg,” “Hand/arm,” “Head excluding mouth,” “Mouth/throat,” and “Torso.” The perception and action strength ratings were displayed on separate screens. The order in which these modalities/effectors appeared was randomized for each item list. A small representation of the body avatar was shown in the instructions and next to each action effector label. No predetermined values were assigned to any of the rating scales. At the bottom of the screen, there was a checkbox labeled “I do not know it” and a “Next” button.

Participants were required to finish the ratings or select the option marked as “I do not know it.” before proceeding to the next item. They had the flexibility to modify their ratings until they finalized and submitted the questionnaire online. The survey was designed to be self-paced and allotted approximately 30 min for completion.

### 2.4 Data exclusion and analysis

All analyses were performed by SPSS. The final item set consisted of a total of 207 words, both in Chinese and English. Each word had data in 11 sensorimotor dimensions. We recruited a minimum sample size of 30 participants for each Chinese and English wordlist in the survey. Initially, the sensorimotor strength ratings for each wordlist were based on the input of 30 to 44 participants. To ensure the data collected met high-quality standards, we thoroughly checked participant performance and item completion. We individually inspected the responses of each participant and deleted any data files where the participant responded with “I do not know it.” The proportion of excluded data was minimal, amounting to 1.9 and 9.1% of individual ratings for Chinese and English items, respectively. Finally, excluding “I do not know it” responses, the average number of participants rating each Chinese word was *M* = 32.79, range of *N* = [24, 44] and for rating each English word, *M* = 29.84, range of *N* = [16, 43]. From the remaining data files of exceptional quality, we calculated Cronbach’s alpha per item list per dimension. The Cronbach’s alpha across all dimensions was 0.770 for Chinese words and 0.767 for English words. These results demonstrate a high level of agreement among the overall responses.

To compare the potential differences between the two languages, we conducted repeated-measures ANOVAs on the variables, with language as a within-subject factor. To investigate the relationship between AoA, frequency, and sensorimotor ratings, we conducted a Pearson correlation analysis. Frequency for the Chinese and English words were obtained from the Corpus of Chinese Language (http://ccl.pku.edu.cn:8080/ccl_corpus/) and Corpus of Contemporary American English (https://www.english-corpora.org/coca/) respectively. To examine the potential impact of sensorimotor grounding on bilingual acquisition beyond frequency, we included ratings of sensorimotor experience in L1 Chinese and L2 English as additional predictors in the conceptual features model, along with log-transformed frequency. To address the issue of multi-collinearity in the regression models, we averaged the 11 dimensions of sensorimotor ratings into a composite score for the measurement of embodied semantics. Additionally, to explore the possibility that the interaction between L1 and L2 sensorimotor experience might account for bilingual AoA, we created an interaction term by multiplying the measures of embodied semantics for L1 and L2. Since interaction and cross-language transfer are likely to occur in a bilingual context, both variables are included in the regression models. Two sets of linear hierarchical regression (Enter) were conducted separately for L1 Chinese and L2 English. Specifically, in the regression models, log-transformed word frequency and mean-centered sensorimotor experience ratings were used as independent variables to predict AoA in the specific language. Frequency was entered as the first step to assess its unique contribution to predicting AoA (the frequency model). After controlling for frequency in the first step, the measures of embodied semantics in L1, L2, and their interaction were entered into the second step the regression model (also known as the sensorimotor model) to estimate the additional variance that can be explained by sensorimotor experience in predicting AoA. To determine the respective contributions of each predictor, we calculated the variance of bilingual AoA uniquely explained by each predictor in Step 2. This involved squaring the semi-partial correlations in the regression models for each predictor.

## 3 Results

To explore the effect of sensorimotor grounding on AoA in bilingual context, we first performed repeated-measures ANOVAs to compare the possible language effect on sensorimotor experience on L1 and L2 English concepts. Table 1 summarized the descriptive and the language group comparison results on the variables. It showed that AoAs for the same set of concepts were different between L1 Chinese and L2 English for the present Chinese-English bilinguals, *F* (1, 206) = 1072.19, *p* < 0.001, partial *ɳ*^2^ = 0.84. The two languages were also different in log frequency, *F* (1, 206) = 46.57, *p* < 0.001, partial *ɳ*^2^ = 0.18, and in the 11 dimensions of sensorimotor ratings: Foot/leg, *F* (1, 206) = 116.14, *p* < 0.001, partial *ɳ*^2^ = 0.36; Torso, *F* (1, 206) = 64.22, *p* < 0.001, partial *ɳ*^2^ = 0.24; Hand/arm, *F* (1, 206) = 55.28, *p* < 0.001, partial *ɳ*^2^ = 0.21; Head, *F* (1, 206) = 63.38, *p* < 0.001, partial *ɳ*^2^ = 0.24; Mouth/throat, *F* (1, 206) = 56.80, *p* < 0.001, partial *ɳ*^2^ = 0.22; Seeing, *F* (1, 206) = 80.93, *p* < 0.001, partial *ɳ*^2^ = 0.28; Hearing, *F* (1, 206) = 147.64, *p* < 0.001, partial *ɳ*^2^ = 0.42; Tasting, *F* (1, 206) = 91.33, *p* < 0.001, partial *ɳ*^2^ = 0.31; Smelling, *F* (1, 206) = 72.05, *p* < 0.001, partial *ɳ*^2^ = 0.26; Touch, *F* (1, 206) = 67.44, *p* < 0.001, partial *ɳ*^2^ = 0.24; Interoception, *F* (1, 206) = 32.78, *p* < 0.001, partial *ɳ*^2^ = 0.14.

**Table 1:** Descriptive data and language group comparison between the bilingual variables.

	Chinese		English		*F*	*p*	partial *ɳ*^2^
	Mean	SD	Mean	SD			
AOA	7.95	1.78	12.48	2.17	1079.19	0.001	0.84
Log Frequency	4	0.79	4.39	0.84	46.57	0.001	0.18
Foot/leg	1.70	0.90	1.33	0.84	116.14	0.001	0.36
Torso	1.89	0.80	1.57	0.80	64.22	0.001	0.24
Hand/arm	2.20	0.93	1.92	0.92	55.28	0.001	0.21
Head	2.96	0.60	2.70	0.57	63.38	0.001	0.24
Mouth/throat	1.77	0.86	1.52	0.84	56.80	0.001	0.22
Seeing	2.86	0.86	2.51	0.88	80.93	0.001	0.28
Hearing	1.83	0.72	1.41	0.69	147.64	0.001	0.42
Tasting	0.99	0.89	0.69	0.79	91.33	0.001	0.31
Smelling	1.09	0.79	0.81	0.72	72.05	0.001	0.26
Touch	1.97	1.00	1.66	0.95	64.77	0.001	0.24
Interoception	3.31	0.49	3.16	0.50	32.78	0.001	0.14

### 3.1 Correlation analysis

To addressing the relationship between AoA and sensorimotor strength in the two languages, the correlation analysis showed that, for Chinese words, there was a negative correlation between AoA and sensorimotor strength across dimensions (*p*s < 0.05), except “Foot/leg,” “head” and “interoception” (*p*s > 0.05) (see Table 2). Frequency demonstrated a significant correlation with various sensorimotor capacities, such as hand/arm (*r* = −0.149, *p* < 0.05), head (*r* = 0.201, *p* < 0.01), seeing (*r* = −0.205, *p* < 0.01), touch (*r* = −0.185, *p* < 0.01), and interoception (*r* = 0.141, *p* < 0.05).

**Table 2:** Pearson correlations between regression predictors.

	Measures	1	2	3	4	5	6	7	8	9	10	11	12	13
1	AOA		−0.350^**^	−0.232^**^	−0.280^**^	−0.245^**^	0.173^*^	−0.217^**^	−0.228^**^	−0.166^*^	−0.291^**^	−0.347^**^	−0.319^**^	0.012
2	Frequency	−0.159^*^		0.014	−0.039	−0.105	−0.008	−0.011	−0.145	0.009	−0.049	−0.067	−0.067	−0.021
3	Foot/leg	−0.111	−0.014		0.869^**^	0.649^**^	−0.325^**^	−0.179^**^	0.512^**^	0.182^**^	−0.057	0.174^*^	0.622^**^	0.010
4	Torso	−0.212^**^	−0.063	0.853^**^		0.723^**^	−0.287^**^	−0.030	0.525^**^	0.191^**^	0.088	0.292^**^	0.721^**^	0.156^*^
5	Hand/arm	−0.273^**^	−0.149	0.668^**^	0.666^**^		−0.517^**^	0.075	0.666^**^	0.121	0.238^**^	0.381^**^	0.872^**^	−0.209^**^
6	Head	−0.010	0.201^**^	−0.080	−0.002	−0.378^**^		−0.020	−0.282^**^	0.231^**^	−0.299^**^	−0.337^**^	−0.499^**^	0.488^**^
7	Mouth/throat	−0.220^**^	0.022	−0.170^*^	−0.063	0.032	0.099		0.096	0.362^**^	0.677^**^	0.532^**^	0.010	0.072
8	Seeing	−0.402^**^	−0.205^**^	0.471^**^	0.468^**^	0.580^**^	0.002	0.022		0.290^**^	0.191^**^	0.367^**^	0.629^**^	−0.361^**^
9	Hearing	−0.191^*^	0.119	0.106	0.118	−0.039	0.536^**^	0.318^**^	0.202^**^		−0.062	0.045	0.016	0.137^*^
10	Tasting	−0.252^**^	−0.076	−0.071	0.021	0.170^*^	−0.061	0.701^**^	0.133	−0.004		0.885^**^	0.267^**^	−0.076
11	Smelling	−0.294^**^	−0.105	0.111	0.164^*^	0.329^**^	−0.083	0.575^**^	0.287^**^	0.063	0.897^**^		0.444^**^	−0.137
12	Touch	−0.316^**^	−0.185^*^	0.588^**^	0.648^**^	0.887^**^	−0.391^**^	−0.011	0.539^**^	−0.139^*^	0.205^**^	0.362^**^		−0.194^**^
13	Interoception	−0.059	0.141^*^	0.196^**^	0.373^**^	−0.065	0.505^**^	0.205^**^	−0.098	0.283^**^	0.169^*^	0.122	−0.005	

The left bottom, for Chinese words; the right upper, for English words; *p* values were based on FDR correction for multiple comparison, *p < 0.05; **p < 0.01.

Contrastively, there was a different pattern of correlation for English words. AoA exhibited a stronger correlation with frequency (*r* = −0.350, *p* < 0.01) compared to L1 Chinese (*r* = −0.159, *p* < 0.05). Additionally, AoA was negatively correlated with most dimensions of sensorimotor strength (*p*s < 0.05), with an exception of a positive correlation with “head” (*r* = 0.173, *p* < 0.05) and no significant correlation with “interoception” (*r* = 0.012, *p* > 0.05). But frequency displayed a significant correlation solely with seeing (*r* = −0.145, *p* < 0.05).

### 3.2 Hierarchical regression analysis

AoA for the 207 concepts in L1 Chinese and L2 English with ratings was submitted to two sets of hierarchical regression models. In the first step, frequency was included as a predictor, followed by the inclusion of the measures of embodied semantics in L1, L2 and bilingual interaction in the second step.

In predicting AoA of L1 Chinese words (Table 3), the first step of the hierarchical regressions included “frequency” as a predictor. This resulted in a significant model, *F* (1, 205) = 5.31, *p* = 0.02, explaining 3% of the variance. In the second step, the inclusion of the 11 dimensions of sensorimotor experience led to a further significant increase in the model’s explanatory power, *F* (4, 202) = 18.80, *p* < 0.001, accounting for an additional 18.8% of the variance. As shown in Table 3, the first step demonstrated that frequency can explain the variance of AoA in Chinese words. When the sensorimotor strength was included in predicting Chinese words, sensorimotor experience also can explain the additional variance of AoA. Relative to frequency, the sensorimotor model explained a greater proportion of the variance of AoA with an increase in adjusted R-squared from 0.02 to 0.22. Importantly, sensorimotor strength from L1 Chinese (*p* = 0.003), L2 English (*p* = 0.006), and their interaction (*p* = 0.03) were significant predictors in explaining L1 AoA. The variance explained by each predictor is as follows: Log Frequency, Δ*R*^2^ = 0.05; Chinese Sensorimotor, Δ*R*^2^ = 0.03; English Sensorimotor, Δ*R*^2^ = 0.03; Interaction, Δ*R*^2^ = 0.02.

**Table 3:** Hierarchical regression models (Enter) in predicting AoA of L1 Chinese concepts (*N* = 207).

Model	Predictor	*Beta*	*t*	*p*	*F*	*df*	*p*	*R* ^2^	Adjusted *R*^2^	*R*^2^ change	*F* change	Sig. *F* change
1	Log frequency	−0.16	−2.3	0.022	5.31	205	0.022	0.03	0.02	0.03	5.31	0.022
2	Log frequency	−0.25	−3.79	0.001	15.78	202	0.001	0.24	0.22	0.21	18.80	0.001
	Chinese sensorimotor	−0.81	−3.04	0.003								
	English sensorimotor	−0.80	−2.80	0.006								
	Interaction: Chinese * English sensorimotor	1.07	2.18	0.030								

When the sensorimotor model was fit into L2 English words. This resulted in a similar pattern of results (Table 4). In the first step of hierarchical regressions, “frequency” was entered in the model, the model was significant, *F* (1, 205) = 28.59, *p* < 0.001, accounting for 12% of the variance; In the second step, when sensorimotor strength was entered in the model, *F* (4, 202) = 20.18, *p* < 0.001, accounting for additional 20% of the variance. Including sensorimotor strengths resulted in an increase in the adjusted R-squared from 0.12 to 0.31. Comparatively, sensorimotor strength from L1 Chinese (*p* = 0.064) and L2 English (*p* = 0.335) were not significant predictors for L2 AoA. Comparatively, their interaction (*p* = 0.018) was a significant predictor in explaining L2 AoA. The variance explained by each predictor was as follows: Log Frequency, Δ*R*^2^ = 0.12; Chinese Sensorimotor, Δ*R*^2^ = 0.01; English Sensorimotor, Δ*R*^2^ = 0.00; Interaction, Δ*R*^2^ = 0.02.

**Table 4:** Hierarchical regression models (Enter) in predicting AoA of L2 English concepts (*N* = 207).

Model	Predictor	*Beta*	*t*	*p*	*F*	*df*	*p*	*R* ^2^	Adjusted *R*^2^	*R*^2^ change	*F* change	Sig. *F* change
1	Log frequency	−0.35	−5.35	0.001	28.59	205	0.001	0.12	0.12	0.12	28.59	0.001
2	Log frequency	−0.36	−6.03	0.001	24.29	202	0.001	0.32	0.31	0.20	20.18	0.001
	Chinese sensorimotor	0.47	1.86	0.064								
	English sensorimotor	0.26	0.97	0.335								
	Interaction:Chinese * English sensorimotor	−1.11	−2.39	0.018								

As anticipated, the regression analysis revealed that both frequency and sensorimotor strengths significantly accounted for the variance in AoA. However, it should be noted that other factors may also have an influential role in bilingual concept acquisition as the larger portion of AoA was unexplained.

## 4 Discussion

### 4.1 The Aim of our study

A large body of evidence has suggested a significant relationship between sensorimotor grounding and the acquisition of concepts in L2 literature (Sheikh and Titone, 2015; Norman and Peleg, 2021; Guan and Meng, 2022; Tai, 2023). However, as argued above, conflicting results prevail regarding the extent of the grounding effect on the L2 context (Chen et al., 2020; Norman and Peleg, 2021). The present study aimed to investigate whether sensorimotor grounding can explain the acquisition of bilingual words, independent of their frequency. Bilinguals were asked to assess the extent of sensorimotor involvement in the comprehension of both L1 Chinese and L2 English words.

### 4.2 Sensorimotor grounding explaining AoA beyond frequency

When addressing the first research question regarding whether sensorimotor grounding can explain AoA of word concepts beyond frequency in the bilingual context, the correlation findings indicate that various aspects of sensorimotor experience manifest a stronger association with AoA in L1 Chinese. Conversely, frequency exhibits a greater correlation with AoA in L2 English. The regression analysis conducted for both L1 Chinese and L2 English confirms incorporating sensorimotor experience explained additional variance in AoA, even after accounting for frequency. This substantiates the notion that the sensorimotor aligns more appropriately with the existing data compared to the frequency models, supporting the proposition that the level of grounding concepts in sensorimotor experience impacts AoA beyond mere frequency.

The inclusion of sensorimotor experience significantly improved the fit of the regression models in both L1 Chinese and L2 English. However, it is important to note that a substantial amount of variation remains unexplained. This is due to the omission of semantic features such as concreteness (Brysbaert et al., 2013) and imageability (Bird et al., 2001), which have been shown to impact AoA. The unexplained variance is likely contributed to these factors (Thill and Twomey, 2016). Therefore, it is not to claim that the present model comprehensively accounts for all factors influencing AoA in L1 Chinese or L2 English. Nevertheless, it is suggested that linguistic factors such as input frequency, alone cannot fully explain the acquisition of concepts. The incorporation of sensorimotor experience enhances the fit of the regression models in both L1 Chinese and L2 English, indicating that a mechanism for grounding in rich sensorimotor experience is necessary for concept acquisition. While sensorimotor ratings provide a good starting point for measuring embodied experience, it is important to consider the diverse relationships that a concept may have with multiple factors in various modalities. Therefore, further research is needed to validate other embodied experiences in the context of L2 acquisition.

Overall, this study represents one of the pioneering efforts to analyze and contrast the impact of sensorimotor experience versus frequency within the bilingual context. Juxtaposing sensorimotor experience and frequency provides a comprehensive understanding of the factors that contribute to the development of bilingual language skills. Findings illuminate the relative importance of sensorimotor experience compared to frequency in bilingual language development. The study enhances our understanding of bilingual language abilities and contributes to the advancement of research and practice in the field of bilingualism.

### 4.3 Comparing the contribution of sensorimotor to AoA between L1 Chinese and L2 English

In addressing the second research question, we compared the L1 Chinese versus L2 English on the correlation patterns and fit of frequency vs. sensorimotor in regression models. Overall, the correlation patterns imply that frequency has a greater significance in the acquisition of L2 English compared to L1 Chinese, whereas contextual information exhibits a stronger link with L1 Chinese word learning (see Table 2). The fit of the regression models further affirms that frequency explains a larger portion of the variance in predicting AoA of L2 English (*R*^2^ Change = 0.12) compared to L1 Chinese (*R*^2^ Change = 0.03). The analysis of the unique contribution of each predictor highlights the significance of language input frequency in the acquisition of words in both L1 and L2, and language input frequency accounts for a greater proportion in explaining the variance of AoA in L2 English compared to L1 Chinese. Critically, in line with our predictions, sensorimotor strength explained less variance in predicting AoA of English words compared with Chinese words. These findings are consistent with previous studies that suggest L2 is more likely a disembodied nature. The differences in the embodiment may be attributed to the influence of acquisition styles, particularly natural acquisition in L1 versus formal instruction in L2, on perceptual representations (Chen et al., 2020). A formally learned L2 is less connected to real-life experiences and thus processed in a less embodied manner relative to a naturally acquired L1.

Considering the relative contribution of frequency and sensorimotor strength in bilingual word acquisition, we argue that theories of embodied cognition provide a structure within which we can investigate the mechanisms underlying grounding impact on bilingual learning. This approach acknowledges that language learning is not merely a process of language input but also involves sensorimotor interactions with the environment. Therefore, the question of how bilinguals acquire words can be reformulated.

For one, sensorimotor experiences have relatively more importance in the early learning of L1. Experience is an essential requirement for language competence as it is closely tied to the ability to effectively engage with the world and communicate with others (Buccino and Mezzadri, 2015). This understanding helps us recognize the crucial role that physical experiences and bodily interactions have in shaping language learning during a child’s early developmental stages. The embodiment of language through sensory experiences aids children in developing a more comprehensive understanding of their L1.

For another, L2 learning is less embodied and more reliant on the frequency of language input. Despite the less contribution of sensorimotor experience to L2 learning, the present findings point to the importance of interaction between L1 and L2 embodied semantics in predicting L2 AoA (Table 4). The evidence highlights the embodied semantics is intertwined with both languages. The embodied semantics also hold promise for utilizing sensorimotor grounding to scaffold L2 learning in formal educational settings (Eskildsen and Wagner, 2013). The multimodal perspective on language and the translanguaging strategy are becoming increasingly popular in multilingual settings (Jusslin et al., 2022; Tai, 2023). The former places emphasis on incorporating physical and sensory involvement in the process of language acquisition. The latter encourages the crossing of boundaries not only between specific languages but also among linguistic and other symbolic modes of communication.

Regarding the role of sensorimotor experience across languages, the negative Beta values in Table 3 show that sensorimotor experience from Chinese and English have nearly equal contributions to AoA in L1 Chinese (Chinese sensorimotor: −0.81; English sensorimotor: −0.80), suggesting that the more sensorimotor involvement in both languages, the earlier individuals acquire words. One striking finding is that the sensorimotor interaction between L1 and L2 has a positive Beta value (1.07) in predicting L1 AoA (Table 3). This contrasts with the negative value (−1.11) in predicting L2 AoA (Table 4). Given the main effects of sensorimotor shown in Table 4, the interaction suggests that for every unit that Chinese sensorimotor increases, the slope of L2 AoA on English sensorimotor is expected to decrease. Hereby, we tentatively argue that the stronger the L1 sensorimotor contribution, the less L2 sensorimotor strengths would be summoned to learn the same concept in L2. However, the nature of the interaction is less characterized. Future research is necessary to address the mechanism of sensorimotor interaction in bilingual learning.

### 4.4 Limitations

The present study integrates measurements of frequency and sensorimotor elements to elucidate the variances of AoA in bilingual word learning. Despite the findings, there are several limitations in the study. One limitation pertains to the failure to classify words into different categories due to the limited number of words. Thereby, we disregard the potential disparity in sensorimotor information for words with different parts of speech. Further investigation is necessary to incorporate a broader range of bilingual words with various categories. Future research is to warrant how word categories modulate the impact of sensorimotor experiences on AoA. Another limitation arises from the omission of various semantic features like concreteness and imageability (Ma et al., 2009; Lynott et al., 2020), which potentially exert influence on AoA, but were not accounted for within the regression models. As a follow-up, it is imperative to collect supplementary data encompassing other linguistic, semantic, and sensorimotor features, to delve into the multifarious determinants that contribute to AoA. It is of note, that there is a need for the development of additional measures that explicitly address sensorimotor experience in L2. Although there are currently some corpora of data that provide measures of sensorimotor experience in L1 English (Thill and Twomey, 2016), to the best of our knowledge, no such data are available for Chinese-English bilinguals. Therefore, additional work should be focused on amassing linguistic, semantic, and sensorimotor metrics that can be used to unravel the nature of Chinese-English bilingual word processing and learning. Finally, a portion of the participants recruited could be classified as trilinguals, possibly with English as their L3 rather than their L2. Consequently, the present findings may be subjected to the generalization issue.

### 4.5 Summary

The present study examined the impact of sensorimotor strength on the acquisition of concepts in the context of Chinese-English bilingualism. Two key findings can be inferred from the study. Firstly, sensorimotor strength can explain additional variance in predicting AoA of learning L1 Chinese and L2 English, even after controlling the effect of frequency of language input. This underscores the significance of sensorimotor grounding in bilingual learning. Secondly, due to substantial differences in AoA for the same concepts in L1 Chinese and L2 English, the degree of sensorimotor strength predicted AoA varies between the two languages. In L1, sensorimotor experience accounted for a greater portion in explaining the AoA variance, whereas L2 seems to be less embodied. These results indicate that the acquisition of bilingual concepts should consider the incorporation of appropriate sensorimotor experiences, in addition to frequent language input.

## Data availability statement

The datasets presented in this study can be found in online repositories. The names of the repository/repositories and accession number(s) can be found below: OSF, https://osf.io/kpqjv/.

## Ethics statement

The studies involving humans were approved by the Experiment Ethics Committee at the School of Foreign Studies, University of Science and Technology Beijing. The studies were conducted in accordance with the local legislation and institutional requirements. The participants provided their written informed consent to participate in this study.

## Author contributions

JX: Conceptualization, Data curation, Formal analysis, Funding acquisition, Investigation, Methodology, Project administration, Software, Supervision, Writing – original draft, Writing – review & editing. DX: Conceptualization, Investigation, Methodology, Project administration, Validation, Writing – review & editing. XL: Investigation, Project administration, Resources, Validation, Writing – review & editing. ZN: Investigation, Resources, Writing – review & editing. FM-R: Conceptualization, Methodology, Supervision, Writing – original draft, Writing – review & editing.
